# Satellite DNA: An Evolving Topic

**DOI:** 10.3390/genes8090230

**Published:** 2017-09-18

**Authors:** Manuel A. Garrido-Ramos

**Affiliations:** Departamento de Genética, Facultad de Ciencias, Universidad de Granada, 18071 Granada, Spain; mgarrido@ugr.es; Tel.: +34-958-249-710

**Keywords:** satellite DNA, Next-Generation Sequencing (NGS), high-throughput in silico analysis, satellite DNA evolution, satellite DNA transcription, satellite DNA function, heterochromatin, centromere, telomere

## Abstract

Satellite DNA represents one of the most fascinating parts of the repetitive fraction of the eukaryotic genome. Since the discovery of highly repetitive tandem DNA in the 1960s, a lot of literature has extensively covered various topics related to the structure, organization, function, and evolution of such sequences. Today, with the advent of genomic tools, the study of satellite DNA has regained a great interest. Thus, Next-Generation Sequencing (NGS), together with high-throughput in silico analysis of the information contained in NGS reads, has revolutionized the analysis of the repetitive fraction of the eukaryotic genomes. The whole of the historical and current approaches to the topic gives us a broad view of the function and evolution of satellite DNA and its role in chromosomal evolution. Currently, we have extensive information on the molecular, chromosomal, biological, and population factors that affect the evolutionary fate of satellite DNA, knowledge that gives rise to a series of hypotheses that get on well with each other about the origin, spreading, and evolution of satellite DNA. In this paper, I review these hypotheses from a methodological, conceptual, and historical perspective and frame them in the context of chromosomal organization and evolution.

## 1. Introduction

Eukaryotic genomes are composed of a large amount of different classes of repetitive DNA sequences, either dispersed (mainly transposons and retrotransposons as well as retrotransposed sequences and some protein-coding gene families) or arranged in tandem (ribosomal RNA, protein-coding gene families, satellites, telomeric DNA, centromeric DNA) [1,2]. Repetitive DNA would explain the C-value enigma [3]. Thus, there is not a correlation between C-value (the DNA content) and the organism’s complexity. Differences in genome size between species, even closely related species, are at times thousands of orders of magnitude [4,5,6,7,8,9]. Among repetitive sequences, transposable elements (TEs) are mostly responsible for the pronounced differences between genomes. Thus, for example, TEs represent the 45% of the human genome [10], the 52% of the opossum genome [11], or the 85% of the maize genome [12]. However, in addition to TEs, satellite DNA (satDNA) also contributes greatly to genomes. Satellite DNA families are organized in large tandem arrays of highly repetitive non-coding short sequences.

There are several considerations to do concerning the distribution of satDNA families among species and their contribution to the C-value:

(i) Different satDNA families may be present in one species. For example, there are up to 15 families in *Pisum sativum* [13], 62 families in *Locusta migratoria* [14], or 9 satDNA families within the human genome [15,16]. However, there are usually one or a few predominant satDNA families in each species [13,14,15,16,17,18,19]. For example, the most abundant satDNA family within the human genome, the centromeric α satDNA, represents more than half of the total content of satDNA in the human genome [10,15,16]. On the other hand, the number of satDNA families must be taken relatively at times, as some of these satDNA families might be related to each other or on the contrary comprise different subfamilies of the same satDNA family.

(ii) Related species may share a set or a library of satDNA families. As mentioned in the previous point, among the families that make up the library is typical for each species to have one or a few predominant satDNA families. For example, different satDNA families are shared by the genomes of different species of the beetles of the family Tenebrionidae, each species having one or two predominant major satDNA families [17,18,19], as occur for the major satDNA of *Tribolium castaneum* (TCAST1) [20,21].

(iii) Concerning a particular satDNA family, this may be species-specific, which is in accord with the accepted view that satDNA is the evolutionarily fastest part of the genome (see below). However, in addition to species-specific satDNAs [22,23], there are also satDNA families shared by several related species within a genus [24,25,26,27,28], by the whole group of species composing a genus [23], by several genera from a family [29], by a whole family [30,31,32,33], by several families [34], or by a whole order [35,36,37,38]. Furthermore, a satDNA family might be preserved in various taxa of a phylum [39,40]. 

(iv) SatDNAs can represent a large proportion of the genome of one eukaryotic species, but not always. In plants, for example, satDNA can represent between 0.1% and 36% of the genome [41,42,43,44,45,46,47]. There is also a wide range of variation in satDNA content within the animal kingdom with values differing between less than 0.5% and more than 50%, both in invertebrates and vertebrates [15,16,17,18,19,20,21,48,49,50,51]. In both cases, plants and animals, the differences in satDNA content might significantly contribute to considerable genome-size differences between related species or even between cytotypes of the same species [9,44,45,47,51]. At times, these differences between species are due to the differential amplification of one particular satDNA while other times the differences are mediated by the sum of all satDNA families from each species. These are two examples among many of them: (a) the genomic content of the FriSAT1 satDNA varies greatly (0.1–36%) between different species of the plant genus *Fritillaria* [45]; (b) it is estimated that *Drosophila simulans* has 5% of the genome composed of satDNA but only the 0.5% of the *Drosophila erecta* genome is satDNA (reviewed in [9]). However, there are species such as *Drosophila melanogaster* with over 20% of the genome or *Drosophila virilis* with nearly 50% of the genome composed of satDNA [9].

Undoubtedly, the data that we have about the proportion of satDNAs in the eukaryotic genomes are fragmentary. Firstly, the data on satDNA genomic content was many times based only on rough estimations and, most times, the total abundance of satDNA in most eukaryotic genomes was probably higher than the estimated one. For decades, rough estimations of satDNA content within genomes have been used by indirect quantification methods (see below). Secondly, until a few years ago, satDNAs were underrepresented in genomic projects (see below). Further, the main procedure to identify and isolate satDNA families from the eukaryotic genomes during the last four decades (genomic restriction digestion and electrophoresis yielding a ladder pattern) is a biased method for the identification of usually one or, sometimes, a few satDNA families of a given genome but not to the detection of the complete set of satDNA families from one species. Thus, for example, in addition to satDNA families identified by conventional methods, several other satDNAs were identified after a genomic approach in several species [9,13,52]. 

Since its discovery more than 50 years ago, satDNA is still today one of the most intriguing, but also fascinating, parts of the genome. However, things are changing because the methodology in the analysis and the knowledge of genome are also changing. The classic review by Maxine F. Singer [53] put the accent on a new way of studying satDNA, which was a starter point for many researchers who opened up the ways for the decipherment of satDNA. Singer emphasized the conception of how methods change through time and how these changes lead to changing concepts. Today, after 35 years, new methods of genomic analysis are also provoking conceptual changes on the topic of satDNA. This review tries to summarize all the advances in the knowledge of satDNA, its organization, its function, and its evolution. Today, we have a better perspective on the knowledge of these aspects. The review focuses on the approach to the organization, to the function and to the evolution of satDNA also from this perspective of the evolution of all we have been learning during the years.

## 2. Changing Methods

The existence of repetitive DNAs in the genomes of eukaryotes was first unveiled in 1961 by Kit et al. [54] and by Seuoka et al. [55]. These pioneering works revealed that genomic DNA from mouse exhibits two buoyant DNA bands in density-gradient ultracentrifugation of DNA using cesium chloride. One of these two bands, the minor component, representing about 10% of the genome, was called satellite DNA. The DNA of this band underwent higher rates of renaturation than the rest of the nuclear mouse DNA or than the simplest genomes composed mostly of unique sequences, a proof that the satellite peak of the mouse genome was composed of repeated nucleotide sequences [56,57,58,59]. Britten and colleagues [56,57,58,59] developed the Cot analysis, based on the principles of DNA renaturation kinetics according to which the DNA sequences re-associates at a rate that is directly proportional to the number of times it occurs in the genome. They demonstrated that a variable proportion of every eukaryotic genomic DNA is composed of repetitive elements and that eukaryote genome sequences can be divided into highly repetitive, moderately repetitive, or single-copy classes of DNA sequences according to their reiteration frequency. Cot analysis thus represented a powerful tool by which highly repetitive, moderately repetitive and single/low-copy DNA can be selectively and efficiently fractionated, cloned, and characterized determining their complexity, composition and abundance [60]. 

**Figure 1:**
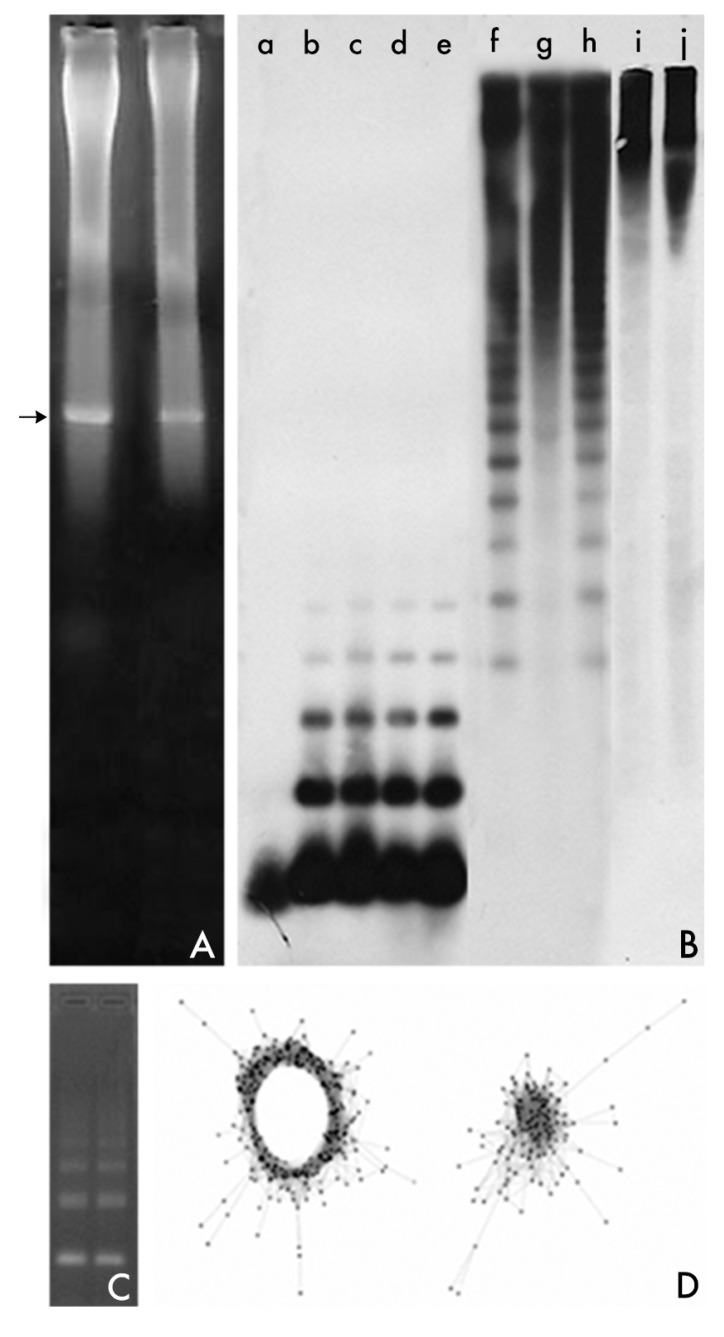
(**A**) After electrophoresis of restriction digested genomic DNA, a distinct prominent band (arrow) against the background smear might be unveiled apparent which contains single fragments corresponding to the individual monomeric members of a satellite DNA (satDNA) family. (**B**) Southern blot hybridization patterns using satDNA probes against genomic DNA digested with different restriction enzymes. The occurrence of a restriction site for a particular enzyme leads to the complete digestion of an array which leads to a band of monomeric units (type A digestion, [53,61]). However, it is common that part of the array remains as a ‘ladder’ of oligomers of the repeat unit since some units have lost the restriction site by mutation (**a**, **b**, **c**, **d**, **e**). On the contrary, mutation may lead to the appearance of occasional restriction sites in some repeats within the array, which is observed as a type B digestion (**f**, **g**, **h**) [53,61]. Patterns of undigested satDNA are found in lines **i**, **j**. (**C**). Ladder-like patterns of satDNA amplification by polymerase chain reaction (PCR). (**D**). Examples of repeat clusters of satDNA visualized in the form of graphs where nodes represent sequence reads and edges connect reads with sequence similarities as obtained using RepeatExplorer [74,75].

These two methods dominated analysis of repeated DNA sequences during the 1960s and 1970s [53]. These methods were substituted by the isolation from restriction endonuclease treatment of genomic DNA. The method simplified and popularized the study of satDNA, assisted to separate satDNA families from other repetitive sequences, and aided in uncovering cryptic satDNAs [53]. Following digestion of genomic DNA with a restriction enzyme and electrophoresis of the DNA fragments generated on agarose gels, satDNA sequences are revealed as a prominent band against the background smear [53]. In this way, the individual members of a repeated set of sequences are available for cloning after the prominent band is excised from the agarose gel, the agarose slice is melted and the DNA purified (Figure 1). These DNA fragments isolated from agarose gels have been found to be the source of numerous studies of satDNA families from a large number of eukaryotic species. 

Roughly, quantification and global genomic organization may be addressed by using dot-blot and Southern blot hybridization techniques (Figure 1). In dot-blot analyses, defined amounts of total genomic DNA as well as the defined amounts of the unlabeled probe (repeat units of the satellite DNA family) are denatured and immobilized on nylon and hybridized with the labeled probe. The relative amounts of the satDNA family in the genome are estimated then by comparisons between densitometric scans of hybridization signals in genomic DNAs and those obtained for the reconstruction standards. The dot-blot hybridization was also commonly used for the detection of satDNA families of one species within the genomes of related species, a method that even permitted the use of this technique in an applied way for the establishment of phylogenetic relationships by the cladistic association of species in monophytletic groups. The organization of a repetitive DNA family in a genome may be analyzed by Southern blot hybridization (Figure 1). Three typical patterns can be observed after hybridization [53,61]. Southern blot hybridization patterns give a rough indication of sequence variation within and between species and contain certain useful phylogenetic signals [62].

**Figure 2:**
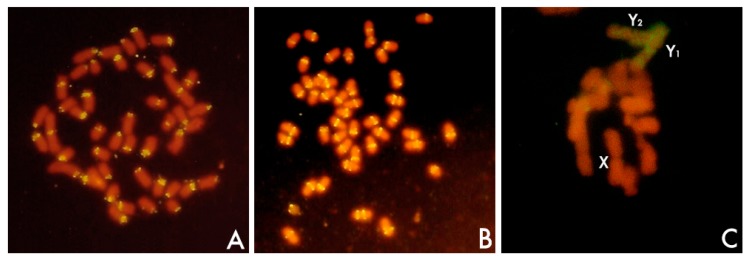
Fluorescent in situ hybridization (FISH) using satDNA probes. Probes were labeled with digoxigenin-dUTP and detected with antidigoxigenin FITC-conjugate. FITC (Fluorescein IsoTioCyanate) is a fluorochrome derivative of fluorescein which fluoresces yellowish-green. In these pictures, yellowish-green signals correspond to detected probes while chromosomes are counterstained with ethidium propide (red). (**A**) EcoRI satDNA is the main component of the centromeres of the chromosomes of the fish species of the Sparidae family. This picture shows the detection of the hybridization signals of EcoRI repeats on the centromeres of all the chromosomes of *Diplodus bellotti* [30]. (**B**) DraI satDNA is located in the subtelomeric region of the chromosomes of some sparid species [29]. This picture shows the detection of the hybridization signals of DraI repeats at the subtelomeric region of many of the chromosomes of *Pagrus auriga*. The chromosomes of this species are all acrocentric. Also look for the presence of some interstitial loci in some of its chromosomes. (**C**) RAE180 satDNA is highly amplified in the Y chromosomes of males (XY_1_Y_2_) of the dioecius plant species *Rumex acetosa* [25,26,28]. This picture shows the detection of the hybridization signals of RAE180 repeats which are widespread in the major part of the two Y chromosomes. Sex chromosomes are indicated.

In addition to Southern blot hybridization, the developmet of in situ techniques of hybridization was a breakthrough in the characterization of a satDNA (Figure 2). First attempts using radiolabeled probes were promising [63] and revealed that satellite sequences were located in the heterochromatin, in this case, the centromeric heterochromatin of mouse chromosomes. Notwithstanding, the real revolution, popularization, and potential of this technique came from the use of nonradioactive methods [64] and above all with the advent of fluorescent in situ hybridization (FISH), a powerful tool that may combine the use of different labeled probes with different fluorochromes [64,65].

The sequence of a satDNA family can be obtained by the entire set of monomeric units purified from melted excised agarose gels. Unambiguous consensus sequences are frequently obtained with uncloned sets [53,66,67]. Although this method allowed determining a rough estimate of satDNA variation [66,67], the sequencing of cloned members of the set has been the common procedure during the last four decades. The advent and the advantages of the polymerase chain reaction (PCR) technique extended its use for the isolation of repeats of a satDNA family from one species or from several related species, both by the selection of one primers pair or the combined use of several primers pair in order to uncover the whole variant type sequences found in each genome [68]. Sequencing of repeats of satDNA families obtained by both cloning and PCR, combined with Southern blot and in situ hybridization, has provided a wealth of information about the organization and location of satDNA repeats, the length and copy number of satDNA repeats, the satDNA repeat variability, the functional role of satDNA sequences, and the satDNA evolution as well as its (moderate) use in phylogenetic analysis and in taxonomic studies [1,2,41,69,70,71,72]. 

Meanwhile, over decades, satDNA sequences were left out of the great genome projects since difficulties arose in the assembly of contigs containing repeat sequences. It is obvious that a genomic perspective in the isolation and analysis of satDNA repeats would overcome the bias of the sequence data obtained by cloning or PCR methods. Thus, for example, there are many monomers that escape to cloning when they are isolated from prominent bands, which were excised from agarose gels, containing the monomer subset obtained after complete digestion. In addition, this procedure omits the cloning of the multimers resulted from undigested repeats lacking the site for the restriction enzyme utilized. This bias in the isolation procedure would be maintained in subsequent PCR experiments which use primers designed from information gathered by the aforementioned procedure. Furthermore, there are added difficulties when a satDNA family is poorly represented in a genome. Obstacles also arise when one is dealing with species having high genome size or with small quantities of satDNA. Further, one may raise the question of how many satDNA families are present within a genome. Some of these families may be overlooked in a routine examination conducted using restriction enzymes for their identification. In this context, the integration of Cot analysis, DNA cloning, and high-throughput sequencing was proposed as an attractive methodology that facilitates genome characterization [60].

Thus, satDNA analysis has found an ally in high-throughput sequencing of genomes using Next-Generation Sequencing (NGS). NGS and high-throughput in silico analysis of the information contained in NGS reads have transformed the study of repetitive DNA [41,73]. An efficient pipeline called RepeatExplorer [74,75] has been developed which allows for the de novo identification of repetitive DNA families in species lacking a reference genome [13,73,74,75,76]. RepeatExplorer follows a similarity-based read clustering approach that allows detection of repetitive sequences, which are identified as groups of frequently overlapping sequence reads in all-to-all read comparisons [13,73,74,75,76]. The clustering procedure employs graph-based methods that transform read similarities to a virtual graph, where reads are represented as nodes and their similarities by edges connecting the nodes. The identification of communities of densely connected nodes allows for the identification of various families of repetitive DNA sequences (Figure 1). The reads within the sequence clusters can be assembled to generate contigs that represent the repeats they contain [13,73,74,75,76]. The combination of NGS and computer analysis favours an in-depth global genomic analysis on the repetitive content of genomes and gives us the opportunity to uncover satDNA families whose isolation was elusive by other methods [8,13,14,43,52,77,78,79,80]. In a further step in the use of RepeatExplorer, Ruiz-Ruano et al. [14] have implemented a bioinformatic toolkit (satMiner) which allows for the identification of satDNA families that are extremely rare in the genome. This pipeline consists of several rounds of RepeatExplorer clustering separated by filtering out the reads containing already known satellites, thus increasing the likelihood of finding new rare satellite families. The method is highly reliable for species with high genome size or with small quantities of satDNA. In a further improvement of RepeatExplorer, Novak et al. [81] have developed Tandem Repeat Analyzer (TAREAN), a computational pipeline for unsupervised identification of satellite repeats from unassembled sequence reads. The pipeline uses low-pass whole genome sequence reads and performs their graph-based clustering. Resulting clusters, representing all types of repeats, are then examined for the presence of circular structures characteristic for tandem repeats. Reads from these clusters are then decomposed to k-mers and fractions of the most frequent k-mers are used for reconstructing representative monomer sequences for each satellite repeat [81].

RepeatExplorer, satMiner, and TAREAN are being extensively used in the analysis of the repetitive DNA content of many plant and some animal species [14,73,82]. The development of all these computer tools is opening new opportunities to uncover the core details of satDNA evolution and to gain insights on the different repeat families making up a given genome, their relative abundance and variability, as well as their roles in different genetic and genomic processes [74,75]. Further, this new perspective greatly contributes to the development of comparative genomics and of phylogenomics [74,75]. However, the graph-based genomics approaches are not the only ones. Wei et al. [9] have developed a method called k-Seek that analyzes unassembled Illumina sequence reads for identify and quantify short tandemly repeating sequences (kmers) of 2–10 bp, repeat lengths that are usual among satDNAs in Drosophila. While the existence of a reference genome is not needed for the use of RepeatExplorer, satMiner, or TAREAN pipelines, or for the use of k-Seek, the existence of a reference genome has facilitated the development of other computer programs for the analysis of the satDNA content of model species such as *Caenorhabditis elegans* or *Tribolium castaneum*. In fact, there are up to 25 conventional programs designed to retrieve tandem repeats (usually, short tandem repeats) from complete or or nearly complete genomes, which were not intended for processing the billions of short reads generated by Illumina or 454 sequencing in an operative time [83,84]. Even so, Subirana and Messeguer [48] have recently developed SATFIND and used it for the identification and analysis of the satellite families in *Caenorhabditis* [49]. Also, Pavlek et al. [85] have recovered the use of the Tandem Repeat Finder (TRF) algorithm [86] through the Tandem Repeats Database (TRDB) [87] for the identification of satDNA families of *Tribolium castaneum*.

## 3. Changing Concepts

The use of the term “satellite DNA” to define highly repetitive DNA sequences organized in tandem arrays was first proposed by Pech et al. [88] and coined and popularized as such by Singer [53], regardless of whether or not these sequences form classical satellite peaks upon isopycnic centrifugation [53]. The term covers a wide diversity of sequences that represent a highly variable part of the eukaryotic genome. SatDNA families differ from each other by their location, their repeat unit length, their abundance, and their nucleotide sequence. Together with satDNA evolution and function, those issues will be reviewed in the following subsections attempting to display how concepts about the satDNA topic have been changing during last decades. 

### 3.1. Nucleotide Sequence Composition and Repeat Organization

In theory, satDNA repeats could be generated from any random sequence. Several satDNA families found in one genome may be related in origin. In these cases, the repeats forming each current different satDNA family were originated from the same shorter sequence [14,25]. However, most satDNA families found within the genome of a species are unrelated in sequence. As a matter of fact, it has been found that current repeats of a satDNA family might be the result of a complex evolutionary process that has led to the current repeat unit from shorter repetitions. Thus, repeat sequences are often composed of direct sub-repeats or motifs that remain as remnants of past events of sequence duplications. Thus, for example, the centromeric satDNAs of tilapias, sparids, or mice are the result of several cycles of duplication and sequence divergence of a basic shorter monomer composed of nine nucleotides [30]. A complex scenario of duplications and expansions was observed in the formation of the HindIII satDNA of sturgeons [89] or in the case of the centromeric satDNA of the wedge sole [90]. Current repeats originated from shorter repeat units were also found in several plant species [25,47,91,92]. 

Further, monomer sequences of some satDNAs might form higher-order repeat (HOR) units. HORs are the result of the simultaneous amplification and homogenization of two or more adjacent monomers [70]. Alpha satellite DNA is the most abundant satDNA of primate centromeres, at least of the simian primates [38]. The alpha satellite of humans adopts two different organizations. One is in the form of monomeric tandem repeats of about 170 bp. The other is organized into HOR structures that consist of multiple (from 2 to 34) head-to-tail basic repeat units [71,93,94,95]. Nucleotide sequence identities vary between 70% and 90% when comparing monomeric repeats of the same multiple unit. However, the similarity is higher than 95% when a monomeric unit of one multiple unit is compared to a counterpart monomer (located at the same position) in another multiple unit [38,93,94]. HOR organization is found at the centromere of human chromosomes while the pericentromeric heterochromatin is composed of single alpha satDNA monomers (50–100% sequence identity), which can coexist with HORs [70,71,96,97,98]. Different chromosome-specific alpha-satellite subfamilies have been described in humans, characterized by HOR organization plus their related monomer sequences [93,94,99,100]. HOR organization was believed to be restricted to hominids but it has been demonstrated that this type of organization is extended to hominoids [101,102,103] and further to the Old World monkeys (Catarrhini) [98,102,103,104,105] and also to the New World monkeys (Platyrrhini) [38,106]. In all characterized species, the monomeric units have a length of 170 bp but during the course of primate evolution this fundamental seeding unit has experienced a number of sequence and structural variations [70]. Old and New World monkeys and gibbons lack chromosome-specific subfamilies [70,101,105]. 

Regular HORs similar to those found in humans, but usually dimeric, have been found in several species of beetles (reviewed in [107,108]). However, the formation of complex HORs, shaped from interspersed and/or inversely oriented monomers and frequently with extraneous sequence elements, is usual (reviewed in [107,108]). Complex HORs have been found in non-human mammals, such as mouse, swine, bovids, horse, dog, and elephant and in insects (reviewed in [107,108]).

Interestingly, several classes of repetitive DNA sequences can be the seed for the formation of a satDNA family. For example, some satDNA families originated from part of the intergenic spacer of ribosomal RNA genes (rDNA) [41,70] or from parts of different classes of TEs [109]. Conversely, tandem repeats have also been found as integral components of TEs [109] and some satDNA were probably derived from tandem repeats present in a hypothetical miniature inverted–repeat transposable element (MITE)-like element [109,110,111,112]. Thus, it has been suggested that mobile elements may be an important source of satDNAs in diverse genomes [109,113,114,115].

### 3.2. Defining satellite DNA

Although location, repeat length, and copy number are features than can be conveniently analyzed separately, I want to connect all of them in the following paragraphs because the variability shown by satDNAs concerning these features is connected also with the definition of satDNA itself. SatDNAs are the main component of the heterochromatin, which is found specifically at pericentromeric and subtelomeric locations of the chromosomes (Figure 2) [1,41,69,70,71]. Additionally, heterochromatin might be found occupying interstitial loci of chromosomes in specific positions of the chromosome arms between the subtelomeric and the pericentromeric regions, especially in invertebrates and plants. Further, heterochromatin might also be found in specific chromosomes such as sex chromosomes (Figure 2), supernumerary chromosomes, or in particular regions of one particular chromosome (see below). SatDNA is also a widespread element nucleating centromeres. However, in addition to satDNA, heterochromatin and centromeres might be occupied also by TEs [41,42,71,109,116,117,118,119,120,121]. Further, simple sequence repeats (SSRs) (also known as single tandem repeats -STRs- or microsatellites) as well as minisatellites are commonly found forming the pericentromeric and the subtelomeric heterochromatin [122,123,124].

Microsatellites are defined classically as tandem repeats of less than 10 bp in length in arrays less than 1 Kb, distributed in loci scattered throughout the genome. Minisatellites are similar but characterized by longer repeats (>10 bp). Most animal and plant satDNA sequences commonly have monomer unit lengths of about 150–180 bp or 300–360 bp, although exceptions to this assumption are far from being exceptional [35,39,41,70]. Even in the event that the repeat length intervals between satDNAs and microsatellites (≤10 bp) and minisatellites (10–100 bp) sometimes overlap, they have been conventionally differentiated by their location. Thus, while satDNAs would be composed of families of tandem repeats located as long arrays at the heterochromatin, micro-, and minisatellites would be proper of euchromatic regions. It has been claimed that simple repeats, when present and organized in arrays of many thousand copies in heterochromatin, they are satDNAs [41]. In fact, today, the term satDNA is applied to any tandem of hundreds to thousands repeat units located in the constitutive heterochromatin. Thus, in a broad sense, megabase-long arrays of repeating units of only few bp should be considered also satDNA [41,125]. Indeed, repeat lengths of ‘classical’ satDNAs vary between a few and several thousand base pairs. Thus, while repeat lengths of some satDNAs are longer than one or two Kb in cetaceans and bovids [35,51] or 5.9 Kb in potato [92], several satDNA of the pericentromeric heterochromatin of human chromosomes are composed of repeats as short or even shorter than the repeat length of common microsatellites [126]. In *Drosophila*, with the exception of three satDNAs, most known satellites are tandem repeats of simple sequences (≤10 bp) [9]. 

Furthermore, recently it has been demonstrated the existence in the euchromatin of loci composed of tandems with repeats of lengths in no way within the range of the micro- or minisatellite classification. Thus, short arrays of satDNAs dispersed along the euchromatin have been described. There are also satDNAs present not exclusively within heterochromatin but also dispersed as single repeats or short arrays within euchromatin [14,21,80,85,127,128,129,130,131]. Similarly, shorter repeats showed the same capability to cluster both in euchromatin and in heterochromatin. In fact, Ruiz-Ruano et al. [14] demonstrated that microsatellites, minisatellites, and satellites show similarities at genomic and cytological levels, with intragenomic dissemination preceding clustering, and claimed that the fact that all kinds of satDNA (micro-, mini-, and satellites) can show non-clustered and clustered states suggests that all these elements are mostly similar, except for repeat length. Also, variable number of tandem repeats (VNTRs) in bacteria showed similar features to non-clustered satDNAs [14].

These new views on the concept of satDNA became possible thanks to the evolving methodology used in the approach of satDNA analysis. These new perspectives lead to the coining of new terms such as those that arise from the high-throughput analysis of genomes. Thus, Ruiz-Ruano et al. have proposed the term ‘satellitome’ for the whole collection of different satDNA families in a genome [14]. The computer analysis of NGS reads allows us to obtain new insights on the origin and evolution of the satellitome, a part of the ‘repeatome’, the term proposed by Kim et al. [82] for the collective set of the repetitive elements of a genome (TEs, tandem repeats, etc.). Inter- and intraspecific satellitome comparative analysis provides information on satDNA organization and evolution as well as chromosome organization and evolution [14].

### 3.3. Principle of the Equilocal Distribution of Heterochromatin

The principle of equilocality of heterochromatin distribution would explain the tendency of heterochromatin to occupy similar location on non-homologous chromosomes [132]. The principle affect to all different sites of heterochromatin—pericentric, interstitial, and subtelomeric—which tend to have different cytogenetic properties [132]. That is, according to this principle, the heterochromatin accumulates at equivalent positions in each chromosome within a genome: pericentromeric and subtelomeric regions, principally. When heterochromatin is built up in an interstiticial region between the centromere and the telomere of a chromosome arm, all of the members of the chromosome set, or a particular subset of chromosomes, may carry heterochromatin at comparable locations [132]. The concept applies to satDNA and assumes that different satDNAs families occupy different equilocal sites. In fact, even in one species with many satDNA families such as *Locusta migratoria*, different satDNAs families displayed similar equilocal distribution across non-homologous chromosomes supporting the principle of satDNA equilocality [14]. Thus, usually, the pericentromeric heterochromatin of one species is composed of a particular satDNA family which is different from the satDNA family found in the subtelomeric region. When several satDNA families are located in one given chromosomal region, pericentromerically for example, they are arranged in differentially located arrays and this arrangement is maintained in all the chromosomes, as occur in mouse minor and major satDNA families ([133] and references herein).

The concept of equilocality assumes that subtelomeric regions are presumptive sites where heterochromatin amplification tends to be initiated, and from which satDNA sequences are spread to interstitial sites in a process known as interstitialization, which may be facilitated by telomere reunion at the first meiotic prophase bouquet [132,134]. Interstitialization has ocurred in different species through the transference of satDNA sequences from the telomeres towards interstitial sites [135]. The subtelomeric pSc119.2 satellite DNA tended to spread towards new interstitial sites during the diversification of the most primitive form of the genus *Secale* towards the most advanced taxa [135]. This highly repetitive sequence has supported events of interstitialization also in *Hordeum* [122]. DraI is a subtelomeric satDNA family shared by several fish species of the Sparidae family (Figure 2). In addition to subtelomeric location, there are several interstitial loci in some chromosomes of these species [29]. However, the process of interstitialization cannot explain alone the formation of new satDNA families in interstitial loci (see below).

There are clear exceptions to the principle of equilocality. Non-equilocal arrangement of a satDNA family could be due to chromosome reorganization such as inversions and/or transpositions or Robertsonian translocations [27]. 

Absence of exchange between non-homologous chromosomes may lead to the independent evolution of satDNAs in two subgenomes, even at equilocal heterocromatic sites, and to the emergence of different satDNA families, as occur in *Sus scrofa domestica*. This species has a bimodal karyotype 12 meta-/submetacentric (Mc) and 6 acrocentric (Ac) chromosomes (Mc and Ac ‘subgenomes’). The centromeres of each ‘subgenome’ differ by having a different satDNA family. This difference was explained by the spatial arrangement of chromosomes in meiotic pachytene nuclei [136].

On the other hand, an exceptional case of non-equilocal distribution of satDNA is found in the larger chromosome of the plant species *Muscari comosum* which is the result of a massive amplification of the *Muscari comosum* satDNA family (MCSAT), constitutes the 5% of the genome, and leads to the characteristic asymmetry of the karyotype of this species [46]. Additionally, the MCSAT repeat family varies between 0.8% and 5% of the genome between species of the subgenus *Leopoldia* of *Muscari*, which largely contributes to the different degrees of asymmetry of their karyotypes [46].

Alteration of the equilocal distribution of satDNAs also included satDNA amplifications in specific regions of particular chromosomes such as B and sex chromosomes as well as microchromosomes. Sex chromosomes have arisen independently several times in several different groups of organisms from a pair of autosomes. Genetic differentiation of undifferentiated non-heteromorphic sex chromosomes leads to the establishment of sex chromosome heteromorphism [137]. Thus, the gradual suppression of recombination between the sex chromosomes is thought to lead to their progressive divergence and to the erosion of the Y chromosome [138] which results in the loss of function of many genes within the Y chromosome and accumulation of distinct classes of repetitive DNAs such as TEs and satDNAs [137,138,139,140,141,142,143]. The human male-specific region of the Y chromosome (MSY) is a mosaic of heterochromatic sequences and three classes of euchromatic sequences [140]. Among the repetitive sequences, these authors found that the heterochromatin of MSY encompasses at least six distinct satDNA sequences. In Orthoptera, the X0/XX sex-determining system is considered modal but eventually, diverse sex chromosome systems involving neo-Y formation evolved several times [144]. In one of these species, *Eneoptera surinamensis*, a high-throughput analysis of the satellitome has revealed that the neo-Y chromosome harbors the highest diversity of satDNAs documented to date, representing 39 distinct families, with seven being exclusive to this chromosome [144]. Several satDNA families were also deeply analyzed in dioecious plant species. Among flowering plants, only a few dioecious plant species have chromosome-mediated sex determination systems [141,142,145]. The most common case is the existence of XX/XY chromosomal complements although there are other alternatives involving more complex chromosomal systems. Sex chromosomes in plants are in general evolutionarily young existing a great variety of situations from non-heteromorphic sex chromosomes to highly differentiated heteromorphic sex chromosomes through several cases of different intermediate stages or degrees of sex-chromosome differentiation [41,141,142,145]. The Y chromosomes of XX/XY_1_Y_2_ species of the genus *Rumex*, but not those of XX/XY species, are heterochromatic and have accumulated a set of diverse repetitive sequences [25,52,146,147,148,149,150,151]. TEs were found in Y chromosomes of *Rumex acetosa* but satDNA prevented their significant expansion [52]. Kejnovský et al. [152] suggested that microsatellites are probably targets for insertions of transposable elements [152]. Microsatellite accumulation on the Y chromosome in *Silene latifolia* also suggested that the spread of microsatellites predates other structural changes that occurred during the Y-chromosome evolution [153]. SatDNA accumulation is occurring even in an early stage of sex-chromosome evolution such as that of *Silene latifolia* [154]. A high-throughput sequence analysis of repetitive DNA revealed generally low divergence in repeat composition between the sex chromosomes in *S. latifolia*, as expected according their relatively recent origin [155]. A comprehensive review on the impact of repetitive DNA sequences on plant sex chromosomes [156] and various papers on the evolution of sex chromosomes in different systems are treated in this volume and I refer to these articles for a broad view of the subject.

Chromosome-specific satDNAs are also found in particular chromosomes such as B chromosomes [157]. Supernumerary chromosomes or B chromosomes are dispensable genetic material found in about 15% of eukaryote organisms, both plants and animals [158]. They are derived from the A chromosome complement from the same (intraspecific origin) or a different (interspecific origin) species [158]. Interspecific origin may be a byproduct of interspecific hybridization [159] or a product of interspecies introgression [14,158,160,161]. Satellitome analysis has been revealed as a powerful tool to shed light on B chromosome origin and evolution in several cases. Thus, for example, the B chromosome in the grasshopper *Eumigus monticola* most likely arose from the proximal third of one autosome through a breakpoint that it could be delimited by the authors, as deduced from satellitome analysis [161]. In addition, two specific satDNAs in the B chromosome could be originated intra-specifically since seeds of smaller satDNA repeats probably involved in their formation were already present in the B-lacking genome, suggesting their massive amplification in the B chromosome after the origin of the supernumerary chromosome [161]. Similarly, A and B chromosomes of *Secale cereale* contained a similar proportion of repeats but differed significantly in composition by an additional massive accumulation of B-specific satDNAs [157]. Some B-enriched sequences are unique to the supernumerary chromosome, but not all of them, which suggest that B originated from A chromosomes [157]. Rye satDNA families including those clustered on the B chromosome are transcriptionally active. These B transcripts could have a function as scaffold RNA in the organization and regulation of Bs [162]. Indeed, B chromosomes, though initially inert elements, possess transcribed sequences and even transcribed pseudogenes and protein coding genes [162,163,164,165,166]. For a review of this topic, see the paper of Ruban et al. [167] in this volume. The same as B chromosomes, supernumerary chromosome segments have also been described, some of them being heterochromatic and plenty of satDNA sequences [168,169,170], but neither all supernumerary chromosomes nor all supernumerary chromosome segments are heterochromatic [41].

Bimodal karytoypes composed of macro- and microchromosomes are frequent in birds and reptiles. In some cases, there are microchromosome-specific satDNAs suggesting an independent evolution of these chromosomes with respect to the macrochromosomes [171]. For example, centromeric repetitive sequences have been isolated in struthioniformes, galliformes, and one species of turtle ([171] and references therein). In contrast, Matsubara et al. [171] analyzed three satDNA families in two species of snakes—the habu snake (*Protobothrops flavoviridis*, Viperidae) and the Burmese python (*Python bivittatus*, Pythonidae)—and either satDNA sequences showed no genomic compartmentalization between the macrochromosomes and microchromosomes as was found previously in lizards and skinks ([171] and references therein). On the basis of chromosome number, the sturgeons can be divided into two groups: the first includes those with a diploid number of 120 and the second includes those with a diploid number of about 240–260. Additionally, their karyotypes are characterized by the presence of numerous microchromosomes. The HindIII satDNA family is located in the centromeres of a reduced set of chromosomes which correlates with species ploidy [172]. Thus, diploid species have 8 HindIII centromeres while tetraploids have from 50 to 80 HindIII centromeres. In both cases, there was no distinction between micro- and macrochormosomes [172], but the results suggest the existence of other satDNA families or variants of the HindIII family populating the rest of centromeres. The DBC-150 satDNA family is restricted to a single pair of microchromosomes in species from the *Drosophila buzzatii* cluster [173].

### 3.4. Satellite DNA Evolution

According to the hypothesis developed by Ruiz-Ruano et al. [14], the birth of a satDNA family implies the de novo duplication of a genomic sequence of two or more base pairs that gives rise to a short tandem repeat at a unique genomic location. The formation of this locus can occur by various mechanisms such as, for example, the slippage of one DNA strand during DNA replication or the reinsertion of replicate copies from extrachromosomal circular DNA intermediaries. In a second step, this repetitive family can be disseminated throughout the genome by mechanisms such as transposition or reinsertion of replicate sequences from extrachromosomal DNA intermediaries. At that point, some loci may be amplified locally. Genomic restrictions and natural selection would henceforth place rigid limits on the accumulation of satellite DNA, especially in prokaryotes. In eukaryotes, however, some loci can be amplified locally and exceed the detection threshold of cytogenetic techniques (such as fluorescence in situ hybridization -FISH-) thus becoming a satDNA locus visible by FISH. Local amplification involves a rapid increase in the size of the tandem that could take place, for example, by unequal crossing-over. On the basis of this model, tandem repeats of 15 bp or less may appear by chance in many places in the genome in such a way that both microsatellites and shorter minisatellites can begin their life cycle in the second stage. These shorter repeat units can in turn give rise to longer repeat units through several consecutive cycles of duplication and divergence [25,30,89,90]. Similarly, HORs are evolutionary units of amplification and homogenization of smaller monomers [70].

#### 3.4.1. The “Library” Hypothesis

The hypothesis about the origin of satDNA families of Ruiz-Ruano et al. [14], integrates the “library” hypothesis on the expansion of different satDNA families in different lineages [14,17,70,109,174,175]. Related species may share an ancestral set of different conserved satDNA families, each of which may be differentially amplified in each species. When a satDNA family is amplified differentially in one species, low-copy counterparts of it are found in other related species. Monomer variants comparisons of this satDNA family show high interspecific sequence conservation and absence of species-diagnostic mutations, as found in the beetle *Palorus* [17]. This hypothesis has been proven in insects [17,18,175,176] and plants [26,177,178].

Historically, both transposition and replication of extrachromosomal circles of tandem repeats by the rolling-circle mechanism and reinsertion of replicated arrays [66,67] have been postulated as the main mechanisms for the spreading of satDNA families. However, there was no evidence of such mechanisms as driving forces of satDNA dissemination. Cohen et al. [179,180] found evidence for the rolling-circle mechanism. However, it was not until very recently when the structural and functional liaisons between transposable elements and satDNAs have been found [109]. The data would probe that TEs may act as a substrate for satDNA emergence and mobility [109,114,115,181,182]. Mobile elements may significantly contribute to satDNA evolution by generating a library of tandem repeats that can be dispersed through the genome and in some cases amplified into long arrays of new satDNAs [109]. Šatović and Plohl [114] proposed that onset and spread of tandem repeats can be intimately linked to processes of transposition in more cases than expected previoulsy. Pavlek et al. [85] found that, in addition to other mechanisms, transposition might play an important role in the efficient spread of satDNA in *Tribulium castaneum*. According to Šatović and Plohl [114], interspersed TEs and satDNAs, shape eukaryotic genomes and drive their evolution.

These two mechanisms as well as unequal crossing-over might be the responsible of differential amplification of different satDNA families of the ancestral library found in the common ancestor of different descendant species. This model of evolution of satellite DNA may also explain the differential amplification of the subfamilies of a satellite DNA family [68,183,184]. Thus, HinfI is a subtelomeric satDNA family [185], first isolated in the genera *Carthamus* and *Centaurea* [183,185], conserved in all species of the Centaureinae subtribe [68] and in the genome of the species of the Cardueae tribe [184], the largest in the family Compositae. At least nine HinfI subfamilies were present in the common ancestor of Cardueae, each of which has spread differently in different genera of each of the subtribes of this tribe. 

#### 3.4.2. Concerted Evolution

Once a satDNA family has been spread out throughout the genome, each repeat at every tandem constituting the different satDNA loci of a species could follow an independent evolution freely diverging from one another or, on the contrary, they could follow a cohesive evolution [41,70]. In fact, contrary to what would be expected in the absence of selective restrictions, members of a satDNA family would show a high degree of intra-specific similarity and inter-specific divergence, following a pattern of concerted evolution [41,70]. This cohesive and gradual evolution would be allowed through a two-step evolutionary process called molecular drive [186,187]. According to this model, new variants that appear by mutation in individual units are expanded to the rest of repets by means of mechanisms like unequal crossing-over, transposition, or reinsertion of replicated extrachromosomal forms, which together with gene conversion, are involved in the homogenization of satDNAs. In a second step, the new expanded variants are fixed in a population through sexual reproduction.

A main topic of interest concerning concerted evolution is the extent of population differentiation. Up to the present, interpopulation concerted evolution of satDNAs was detected only in satDNAs of pupfish [188]. No other studies on satDNA divergence at the population level did succeed in identifying population-specific mutations or other population-specific sequence features of satDNAs (reviewed in [189]). Feliciello et al. [189] have found evidence for the existence of population-specific satDNA profiles. In *Tribolium castaneum*, there are two highly similar subfamilies of the *Tribolium castaneum* 2 satDNA (TCAST2). They differ by nine point mutations, each homogenized and fixed within a particular subfamily. Sequences of satellite subfamilies do not differ among *T. castaneum* strains but they were differentially amplified among strains. According to the proposal of Feliciello et al. [189], these results would support the hypothesis that satDNA could act as driver of genome divergence at the population level. Variation and population differentiation in satDNA abundance among lines of *Drosophila melanogaster* have also been exposed by Wei et al. [9]. 

#### 3.4.3. Factors Influencing Satellite DNA Evolution

In the absence of selective and biological restrictions, the concerted evolution rate of a satDNA family depends basically on evolutionary time [190]. In these cases, it may be expected that transitional stages during the propagation of a variant from its onset by mutation to fixation can be clearly classified [191,192,193]. However, concerted evolution depends on several intrinsic and extrinsic factors and in many cases this pattern of evolution is affected and altered in such a way that the differences between species are not always as noticeable as might be expected. Contrary to that, the differences between sequences of the same species might be similar to the differences found between different species. As indicated above, time is important and an elevated number of shared polymorphisms between repeats of two different species might simply indicate a short divergence time between the compared species [190,194]. Not always however, since the rate of sequence evolution of a satDNA family could be slowed or accelerated by the effect of location, organization, and repeat-copy number [26], population and evolutionary factors [33,183], biological factors [195,196,197], or functional constraints [19]. Several examples illustrating these effects are found in the following paragraphs.

##### 3.4.3.1. Chromosomal Location, Organization, and Repeat-Copy Number

Analysis of satDNA in the genus *Rumex* demonstrated that the chromosomal location affects to the evolutionary rate of this type of repetitive sequences because the location might affect to its recombination rate [25,26]. Eurasian and American dioecious species of the plant genus *Rumex* (Polygonaceae) form a monophyletic group which is divided in two phylogenetic clades: one composed of species with a simple sex chromosome system XX/XY and another composed by species with a complex sex chromosome system XX/XY_1_Y_2_, this later derived from an ancestor with a XX/XY system. Analysis of the synaptonemic complexes and the cytogenetic characterization of sex chromosomes of species XX/XY and XX/XY_1_Y_2_, revealed that XX/XY species represent an early stage of genetic differentiation between sex chromosomes. In contrast, species with a sex determination system XX/XY_1_Y_2_ showed an advanced state of genetic differentiation between the X and the Y chromosomes [26,149]. In such a way that the Y chromosomes do not mate with each other, pairing only at one end with each of the X ends in a sexual trivalent. Thus, there is no recombination, or is very limited among the Ys, being very restricted between X and Y [149,198]. XX/XY_1_Y_2_ species have heterochromatic Y chromosomes but not the XX/XY species [149]. Up to seven satDNA families have been characterized in XX/XY_1_Y_2_ species [25,52,146,147,151,168]. One family, the RAE730 family, is unique to the heterochromatic segment of one autosome. The RAYSI family is unique to the Y chromosomes and is therefore not present in females. The RAE180 family is in the Y chromosomes and, in addition, in a small locus of the pair 2 of the karyotype. Therefore, this satellite is present in the genome of males and females, although in females RAE180 is poorly represented [26]. Mechanisms eluding recombination negatively influence concerted evolution. Thus, RAE180 and RAYSI satellites of the Y chromosomes of dioecious species have an evolutionary rate that is half the rate of change of the RAE730 satellite of autosomes [25]. Unlike the other two satellites, RAE180 is also present in dioecious XX/XY species but in a smaller amount and in an autosomal locus, visible by FISH only in some species. Interestingly, comparing XX/XY and XX/XY_1_Y_2_ species, the same satellite DNA (RAE180) in different locations has different rates of evolution [26]. Thus, the RAE180 has rates of evolutionary change three times superior when in autosomes (in XX/XY) species than when in Y chromosomes (in XX/XY_1_Y_2_ species). In addition, RAE180 sequences located in autosomes show a pattern of concerted evolution that is not observed when comparing sequences of the Y chromosomes [26].

Pavlek et al. [85] have found that concerted evolution acts more efficiently on longer than shorter arrays. Thus, it is probable that ancestral variants in low copy number remnants of the library could escape from the homogenization mechanisms [85,197]. In this sense, it has been proposed that evolutionary periods of stasis would keep variability by the reduced action of molecular mechanisms of non-reciprocal exchange which could be a fact for low repeat-copy number [26]. In this context, it is very interesting also that the repeats placed in the center of one array tend to be homogenized more efficiently than those occurring toward the proximal and distal ends of the array [121,199]. While homogenization mechanisms maintain sequence similarity of repeats within the array, they would also provide divergent repeat variants at the array ends which can be amplified and may be used as a source of new satDNAs [200].

Different arrays on the same or in different chromosomes may experience independent homogenization for arrays- or chromosome-specific repeat variants [122,127,131,201]. Thus, the analysis of euchromatic and heterochromatic repeats from 52 arrays showed that the homogenization of satDNA repeats of the 1688 satDNA family of *Drosophila* occurred differentially for distinct genomic regions, from euchromatin to heterochromatin and from local arrays to chromosomes [127]. Further, the lack of chromosome transfer between non-homologous chromosomes may lead to chromosome-specific subfamilies within a genome [202]. Thus reduction or elimination of exchanges between non-homologous chromosomes would give rise to the formation of satDNA subfamilies as occur for RAYSI satDNA in *Rumex* [148], for PIM357 satDNA of the beetle genus *Pimelia* [203], and for satDNA in spiders of the genus *Tetragnatha* [204]. Absence of inter-chromosome exchange may lead even to the independent evolution of satDNAs in two subgenomes even at equilocal sites as occur in *Sus scrofa domestica* (see above). The observed low rates of homogenization of the DBC-150 family might be related to a presumed reduction or suppression of meiotic recombination in the microchromosomes of *Drosophila buzzatii* [173].

##### 3.4.3.2. Population and Evolutionary Factors

Gene flow between taxa reduces their genetic differences, but also leads to increased intra-specific variation. Therefore, contrary to expectations about the model of concerted evolution, in an evolutionary scenario of reticulated evolution, such as that of the evolutionary history of sturgeons [205], we would find similar or even higher levels of intra-specific variation than inter-specific divergence. This is what occurs in the case of the HindIII and PstI satellite DNAs of sturgeons [33,89]. The repeat sequences of these satellites are not grouped by species affinity in phylogenetic analyses, but the sequences isolated from species belonging to the same phylobiogeographic clade appear intermixed. In addition, the following was observed: (a) high intraspecific variability; (b) low levels of concerted evolution between species belonging to the same phylobiogeographic clade; and (c) a high number of shared polymorphisms between distant species belonging to different clades. Facts, all of them, which could be explained under the pattern of reticulated evolution of these species and interspecific hybridization, common in sturgeons [33,89]. In Cardueae, up to nine subfamilies of the HinfI satDNA have been described. Although phylogenetic trees group the sequences by subfamily affinity rather than by specific provenance, when comparing the repeats of the same subfamily, in most cases, the degree of divergence between any pair of sequences is related to the evolutionary distance between the species compared. However, there are exceptions to this rule, which appear when comparing sequences of species of some genera, such as *Centaurea*, in which reticulated evolution has been a key factor in its evolution [68,183,184].

##### 3.4.3.3. Biological Factors

Luchetti et al. [195] found that sexuality acts as a driving force in the fixation of sequence variants within a satDNA family thus generating intrapopulation cohesiveness and interpopulation discontinuities, and that parthenogenesis has a slowing effect on molecular turnover processes. However, spreading of new variants in unisexual specimens by gene conversion events was also observed. Therefore, given enough time, sequence homogenization can take place in a unisexual species. A similar situation is found for satDNAs of Y chromosomes of the plant *Rumex acetosa*. While the Y chromosomes of *R. acetosa* do not recombine, sister-chromatid interchanges would explain gene conversion homogenizing events which should lead to concerted evolution of Y-linked satDNA subfamilies [148], in resemblance to the human Y chromosome [140]. Accumulated data suggest that evolution of satDNA in ants follows the concerted evolution pattern but that this process is slow in relation with other organisms, probably due to the eusociality and haplodiploidy of these insects [197,206]. As explained in Lorite et al. [197], the haploid males of ants lack meiotic recombination and so the mutation rate in males could counteract the effectiveness of the genome turnover mechanisms. According to these authors, this could partially explain the slowdown in the homogenization and fixation processes [197]. On the other hand, they also explain that eusociality hinders random mating and reduces the number of reproductive individuals to a few units, leading to a high level of variability uniformly distributed among related taxa [196,197]. The effect of autogamy apparently leads to a lack of species-specific variants of ATR-2 satDNA in *Arachis* species (Leguminosae) [178].

##### 3.4.3.4. Functional Constraints

SatDNA is one of the most dynamic components of genomes, undergoing rapid changes in array size and sequence composition within a short evolutionary period [41,70]. Expansion and shrinkage of satDNAs contribute significantly to the array-length polymorphism as well as to the replacement of the most abundant variant with a different variant [41,70]. Further, rapid amplification of one satDNA family from the library, rapidly changes any profile of genomic satDNA [17,70]. Thus, satDNA sequences are rapidly evolving sequences that might cause reproductive barriers between organisms and promote speciation [17,71,189,207,208,209,210,211,212]. However, some satDNAs exhibit sequence conservation of part or of the whole monomer sequence for long evolutionary periods [18,19,24,33,34,35,36,37,38,39,40]. Functional constraints might be influencing in the preservation of satDNAs found in beetles, clams, or several plant groups where some monomer sequences might be evolutionarily preferred in comparison with others [18,19,24,39,40,72]. Some monomers may be preferred because of their functional potential and/or simply because particular combinations of nucleotides and structural features of the DNA molecule are favored by homogenization mechanisms [69,70]. However, biological factors might be responsible for sequence conservation [33,72]. Some satDNAs repeats conserve motifs that are remnants of shorter ancestral repeat monomers which led to the current repeats by duplication and divergence [91]. In contrast, sequence motif conservation might be the consequence of selection drive action. For example, in *Schizosaccharaomyces pombe*, small interfering RNAs (siRNAs) involved in heterochromatin formation derive preferentially from the most conserved regions of heterochromatic repeats [213], which suggest that conservation in this case is due to functional constraints [72,213]. The satDNs’ most known motif is the centromere protein B (CENP-B) box, a 17-bp long motif that is preserved in repeats of primate alpha satDNA harboring the centromeres and in the unrelated centromeric satellite of mouse [214,215,216,217]. The DNA binding domain of the CENP-B protein recognizes and binds the CENP-B box in the centromeric alpha satDNA and mouse minor satDNA. The CENP-B protein and the CENP-B box are largely conserved in mammals [214,218,219,220,221,222]. CENP-B protein facilitates centromere formation and plays an important role in the assembly of specific centromere structures in interphase nuclei and on mitotic chromosomes [221]. In humans, CENP-B is involved in centromere functions, such as de novo centromere protein A (CENP-A) chromatin assembly, CENP-A nucleosome stabilization, and fidelity enhancement of chromosome segregation [222]. Contrary to CENP-B, other centromeric proteins, such as CENP-A, essential for centromere identity and function, are not DNA sequence-specific binding proteins (see below). In the next section, I address this and other questions related to the functional roles of satDNA.

### 3.5. Satellite DNA Function

Over time, it has been accepted that satDNA would be composed of non-coding and non-transcribed repeats associated with heterochromatin with no immediate use, “junk” DNA, even worse “selfish” DNA [41,223,224,225]. Further, the role of satDNA sequences in any biological process was discarded. It was intriguing thus the transcriptional activity of some specific tandem repetitive repeats in newts during embryogenesis [226,227,228,229,230]. Therefore, transcripts of satDNA in oocyte lampbrush chromosomes of newts were viewed as a failure of normal transcription termination probably as a result of read-through from upstream structural gene promoters [226,227,228,229,230]. Similar conclusions were obtained from the analysis of the satDNA transcription in lampbrush chromosomes of pigeon and chickens. In these cases, it was proposed that transcription of repeat sequences was related to its genomic organization [231,232]. Nowadays, however, there is evidence for specific roles of satDNA transcripts on gene and genome regulation. In addition, the early view of satDNA as junk or even garbage DNA has evolved. SatDNA repeats form the centromere locus and the heterochromatin of the pericentromeric area [69,70,71]. In addition, other roles for satDNAs have also been suggested such as chromosome organization, pairing, and segregation [69,70,71]. Furthermore, satDNA transcripts may be involved in the assembly of the kinetochore, in the control of telomere elongation, capping, and replication, in the epigenetic regulation of heterochromatin establishment and maintenance, in the transcriptional response during stress and in the modulation of gene expression [72,233,234].

#### 3.5.1. Centromeres and Pericentromeric Heterochromain

The centromere is a critical locus for the perpetuation of the genetic material that regulates chromosome segregation during mitosis and meiosis by assembling and directing the organization of the kinetochore, a proteinaceous complex that attaches chromosomes to the spindle [69,70,71,235,236,237]. On the other hand, pericentromeric satellite repeats are essential elements that stabilize interactions with DNA binding proteins, maintain heterochromatin architecture, sustain kinetochore formation, maintain sister-chromatid cohesion, and drive chromosomal segregation during mitosis and meiosis [2,69,70,71,72,233,238,239]. 

As mentioned before, the centromeres of most eukaryotes are mainly composed of satDNA. However, there are exceptions. The centromeres of the budding yeast *Saccharomyces cerevisiae* span only about 125 bp of a single copy sequence divided in three conserved DNA elements and are assembled into a single Cse4 (a centromeric H3 histone variant, CENH3) nucleosome that captures a single microtubule [71,235,236,237]. These so-called “point centromeres”, constitute the most known exception to the alternative, most common, “regional centromere” consisting of multiple CENH3 nucleosomes that capture several microtubules [71,235,236,237]. The regional centromere of fission yeast *Schizosaccharomyces pombe* is formed of unique or low-copy sequences flanked by heterochromatic regions [71,235,236,237]. In most animal and plant species, the regional centromere is a “satellite centromere” [237]. The centromere contains large arrays of satDNA sequences which, especially in the case of plants, might be interrupted by mobile elements and it is also flanked by heterochromatin [70,71,96,236,237,240]. However, these centromeric satDNA repeats varies substantially among species, conserved at times only between closely related species, and often being species-specific [41,70,71,77]. In fact, cases have been found in which two or more unrelated satDNA can be found at different sets of centromeres within the same species [241,242,243]. Furthermore, there are functional centromeres lacking satDNA. Six of the twelve potato chromosomes have tandem repeat-based centromeres, but five centromeres do not contain a tandem repeat [242]. It has been found that while most chromosomes of the chicken, horse, and orangutan complement are composed of satDNA, some of their chromosomes contain non-repetitive DNA [236,244,245,246,247]. The centromere in these cases resembles that of neocentromeres formed when centromeres are disrupted in humans [236,248]. Thus, functional neocentromeres may lack centromere-specific sequences and heterochromatin both in plants and animals [236,237,249]. Also, only one of the two centromeres containing the DNA elements of functional centromeres is active in dicentric chromosomes [250,251,252]. Beyond, centromeric retrotransposons are important elements of centromeres of a wide range of angiosperm species [117]. In rice and maize, all centromeres have different retrotransposon families [118,120], and retrotransposons are the main component of banana and some wheat centromeres [42,119]. 

All these data prove that DNA sequences alone are insufficient to determine centromere identity and have revealed the importance of epigenetic factors regulating the centromere identity and function. Regional centromeres of both plants and animals are characterized by the presence of a centromere-specific histone H3 variant CENH3 and are organized as euchromatic domains of CENH3, flanked by heterochromatic domains [70,71,236,237,240,253,254]. Within the centromeric chromatin, CENH3 is interspersed with histone H3 dimethylated at lysine 4 (H3K4me2) and associated repeats are hypomethylated with respect to those found in heterochromatin. Conversely, pericentromeric chromatin is enriched for nucleosomes containing histone H3 methylated at lysine 9 (H3K9me), a mark associated with heterochromatin. For example, a typical human centromere is composed of a fraction of HOR α satDNA built from subdomains of nucleosomes containing centromeric CENP-A (the human histone H3 variant CENH3) interspersed with H3K4me2 and the remainder HORs and monomers of α satDNA are assembled into heterochromatin enriched for nucleosomes containing H3K9me [70,71,253]. Similar to human centromeres, the CENH3 nucleosomes of other analyzed species typically occupy only a portion of the satellite repeats and the rest of repetitive elements are embedded in flanking pericentromeric heterochromatin [240,249,254]. The total size of CENH3 domains is similar between different chromosomes independently of their length or the length of satellite arrays [249].

Most centromeric retrotransposons tested to date are actively transcribed and these transcript might have a role in centromere formation, maintenance, and function [117]. Interestingly, many centromeric satDNA are also transcribed. Transcription of pericentromeric and centromeric repetitive sequences seems to have roles not strictly related to heterochromatin establishment but also in maintaining centromere identity, being an important functional component of the centromere/kinetochore complex [72,233,255,256,257]. Therefore, changes in centromeric satDNA transcription may affect chromosome stability and segregation [72,258,259]. There is a growing body of work showing that transcription of centromeric satDNA in long, single-stranded transcripts encompassing a few satellite monomers [260,261,262], contributes to assembly of the kinetochore, being important for the localization of CENP-A as well as of the kinetochore proteins centromere protein C (CENP-C), inner centromere protein (INCENP) and surviving (an INCENP-interacting protein) at the human centromere [234,260,262]. Recently, it has been showed that centromeric RNAs are processed during mitosis, and that recruitment of the splicing machinery to these transcripts is important for kinetochore assembly [263]. However, it appears that recruitment of the RNA processing machinery to the centromere, but not the persistence or even complete synthesis of mature centromeric RNAs, is important for kinetochore assembly [234]. Transcripts of α satellite play an important role in kinetochore formation but also in the establishment of pericentromeric heterochromatin and are indispensable for the proper cell division [257]. SAT III RNA of *Drosophila melanogaster* is an integral part of centromere indentity by binding to the kinetochore component CENP-C, and because is involved in the correct localization of the centromere-defining proteins, CENP-A and CENP-C, as well as outer kinetochore proteins [264]. Long RNAs not processed into siRNA are also characteristic of centromeres of rice (40 nt), wallaby (34–42 nt), mouse (120 nt), and beetles (multiple transcripts of heterogenous size longer than 500 nt, up to more than 5 kb) [72,258,265,266,267,268]. In rice, long transcripts from centromeric satDNA are processed in RNAs of 40 nt and they act together with 21–24 nt long siRNAs that might derive from the pericentromeric portion of the same satellite [265]. Similarly, the centromeric RNAs of wallaby and mouse are processed from longer double stranded RNAs [266,267]. Thus, RNAi together to an alternative mechanism involving longer RNA might be operating in kinetochore establishment [72].

There is still another type of centromere in addition to point and regional centromeres, the holocentromere, which is spread along the entire length of the chromosome. Holocentric chromosomes lack a primary constriction, in contrast to monocentrics, forming kinetochores distributed along the entire chromosome and microtubules can attach along most of the poleward facing surface [269,270]. The holocentric chromosomes have also been called diffuse-kinetochore chromosomes, holokinetic chromosomes, and polykinetic chromosomes [269,270]. Holocentric chromosomes evolved at least four independent times in plants and at least nine independent times in animals [269,270]. The holocentric chromosomes have been found in the monocots families Juncaceae and Cyperaceae and in the genus *Chionographis* (family Melanthiaceae). Not all genera in Cyperaceae and Juncaceae families are holocentric, but there are some of their genera, as *Luzea*, with holocentric chromosomes. Holocentric eudicots are limited to two genera, *Drosera* (family Droseraceae) and *Cuscuta* (family Convulvulaceae), but not all the species of *Cuscuta* have holocentric chromosomes [269,270]. Recently, Neumann et al. [241,271,272] have identified in species of the legume genera *Lathyrus* and *Pisum* the so-called meta-polycentric chromosomes, monocentric chromosomes with multiple centromere domains, representing a putative intermediate between monocentric and holocentric chromosomes. These domains in *Pisum sativum* are almost entirely composed of repetitive DNA sequences belonging to 13 satDNA distinct families and 1 family of centromeric retrotransposons, all of which are unevenly distributed among pea chromosomes [241]. In the animal kingdom, holocentric chromosomes have been found in two phyla, Nematoda in which holocentric chromosomes arose once and Arthropoda in which this type of chromosomes arose at least eight times [269].

Plant holocentromeres have been extensively studied in the Cyperaceae species *Rhynchospora* [273,274]. Holocentromeres of *Rhynchospora pubera* are highly enriched by the “Tyba” satDNA family that occurs as genome-wide interspersed arrays [273]. This satDNA family is centromere-specific and associates with CENH3 [273]. Centromeric arrays vary in length from 3 to 16 kb and are intermingled with gene-coding sequences and transposable elements [273]. In contrast to other satDNAs, Tyba is dispersed in interphase and with the onset of mitotic condensation, linear satDNA structures along all chromosomes rather than clustered blocks are formed [273]. Thus, holocentromeres of metaphase chromosomes in this species are composed of multiple centromeric units rather than possessing a diffuse organization. A cell-cycle-dependent shuffling of multiple centromeric units results in the formation of functional polycentromeres during mitosis. Holocentricity influences the chromosomal organization of different satDNA families in different species of *Rhynchospora* [274]. Thus, while more conserved centromeric repeats revealed linear signals, noncentromeric species-specific satDNA formed distinct clusters along the mitotic chromosomes. In interphase, the centromeric satDNAs appeared dispersed while non-centromeric species-specific satDNAs appeared clustered [274]. No specific repeats have been found to be associated with CENH3 loci in *Luzula elegans* despite previous efforts to find them [270,275]. In fact, CENH3 has been independently lost in four insect lineages that transitioned from monocentricity to holocentricity [237,276].

Holocentric nematodes have a large number of satDNAs, apparently very few evolutionarily conserved, scattered throughout their genome. In contrast, no scattered satDNAs are found in the monocentric nematode *Trichinella spiralis* [48]. Centromere-like satDNAs described in *Caenorhabditis elegans* may accumulate CENH3, are located in regions with high CENH3 affinity, promote kinetochore formation during mitosis, and have sequence features similar to satDNAs of monocentric species [49]. Subirana et al. [49] found that satellites are randomly distributed in domains of either low or high affinity for CENH3, but that all the longest satellites with longest repeat had higher affinity for CENH3. However, these results contrast with those of Gassmann et al. [277] and those of Steiner and Henikoff [278], who found that CENH3 occupies nonrepeated regions. Steiner and Henikoff [278] did find that holocentromeres show a polycentric distribution, with each site containing a single-wrap centromeric nucleosome and that centromeric sites correspond to transcription factor hotspots, which are bound by multiple transcription factors, but which lack their characteristic sequence-specific DNA-binding motifs [237,278], and do not coincide with satellite positions [49]. Gassman et al. [277] did find that CENH3 occupies nonrepeated regions of 10–12 kb dispersed across about half of the genome and is excluded from loci that are transcribed in the germline and early embryo. 

Independently of sequence conservation, satDNAs may stabilize CENH3 nucleosomes. Thus, satDNA would maintain sequence homogeneity crucial for centromere stability and at the same time it can be a source of extremely rapid changes at centromere [70,71]. Stabilization of CENH3 nucleosomes could explain how new satellites arise and populate centromeres and how neocentromeres based on unique sequences can evolve to repetitive centromeres presumably to stabilize them [236,249]. Tandem duplication of a new sequence with a selective advantage for CENH3 stabilization or transposition of existing satDNAs could populate an epigenetically defined neocentromere [242,249]. 

The CENH3 variant is the defining chromatin component of centromeres in most eukaryotes [276]. Notwithstanding the foregoing, species-specific CENH3 proteins have been identified in all eukaryotes investigated so far, including humans (CENP-A), budding yeast (Cse4), fission yeast (Cnp1), *Caenorhabditis elegans* (HCP-3), *Drosophila melanogaster* (CID), and different plant species [71]. The “centromere drive” model proposes the unequal transmission of competing variant centromeres in the asymmetric female meiosis [208,211,279]. Thus, it has been proposed that positive selection for mutations in genes coding for kinetochore proteins that favor the affinity of these proteins (e.g., CENH3) for centromere sequence variants [71,208,211]. Co-evolution of centromeric satDNAs and kinetochore proteins would explain in this way the variety of eukaryotic centromeres and the rapid evolution of both, sequences and proteins, at centromeres [71,208,211,279]. Rapid changes among individuals in the centromere would lead to reduced compatibility of homologous chromosomes in hybrids and ultimately to postzygotic isolation, thus triggering speciation [71,211,280,281].

According to the centromere drive model, adaptively evolving CENH3 has not been detected in lineages having symmetric meiosis [208,220,279,282,283,284,285,286]. This is because evolutionary changes of CENH3 depend also on the chromosomal structure in terms of kinetochore formation [279]. Thus, most studies addressing CENH3 evolution were carried out on species with monocentric centromeres [279,284]. Also, studies were conducted on *Caenorhabditis* that, although they have holokinetic chromosomes in mitosis, form a cup-like kinetochore in meiosis with ambiguous utilization of CenH3 [279,284]. Thus, the centromere drive model has been questioned recently by Neumann et al. [271]. Two paralogous variants of CENH3 were identified in Fabaceae, CENH3-1 and CENH3-2, which originated from a duplication event in the common ancestor of Fabeae species. Subsequently, while the CENH3-1 gene was lost or silenced in the lineage leading to *Vicia* and *Lens*, having monocentric centromeres, CENH3-1 and CENH3-2 have been retained in the polycentromeric species of *Pisum* and *Lathyrus*. Both genes appear to have evolved under purifying selection and produce functional CENH3 proteins which are fully colocalized [271]. These results are contrary to the predictions of the centromere drive model. Indeed, Zedek and Bureš [279], analyzing CENH3 isoforms in 13 *Luzula* species did not detect positive selection, but on the contrary they found relaxed selection on CENH3. These results have been interpreted from an evolutionary point of view in such a way that holocentromeric chromosomes might have evolved as a defense, suppresing centromere drive [279,287]. Thus, it is possible that holokinetism has evolved only in lineages with asymmetric meiosis where it might be a necessary defense against centromere drive, while in lineages with symmetric meiosis such a defence has no purpose [287].

#### 3.5.2. Subtelomeric Heterochromatin

The telomeres protect chromosomes from degradation and repair activities and prevent chromosome shortening that results from replication of the end of the linear chromosomes [288,289]. Different telomere-specific proteins build telomeres to accomplish these functions [288]. Concerning DNA sequences, telomeres are organized in an ancestral conserved structure based in the repetition of short tandem repeats of about 6 bp: 5′-TTGGGG-3′ (*Tetrahymena*), 5′-TTAGG-3′ (insects), 5′-TTAGGG-3′ (vertebrates), 5′-TTTAGGG-3′ (plants). However, there are some exceptions to this general rule. The vertebrate telomere was also found in marine invertebrates [290]. Many species of Asparagales have telomeres composed by the human-type TTAGGG repeats instead of the plant-type TTTAGGG repeats [291,292]. In *Cestrum* (Solanaceae), the telomeres have the TTTTTTAGGG motif [293]. Despite some variation, the human-type repeat appears to be the ancestral and the most common telomere repeat in eukaryotes [294]. However, alternative motifs have been replacing it along the evolutionary time of diverse eukaryotic lineages [294]. 

Even in the event that telomeric repeats are not included within the category of satDNA, they share many similarities with satDNA sequences and are intimately related with subtelomeric satDNA repeats. However, they are originated and amplified by a different mechanism depending on telomerase, a reverse transcriptase that adds telomeric DNA to telomeres using associated RNA as a template.

Notwithstanding, there are exceptions to this general rule. Thus, *Drosophila* telomere maintenance is different and it is based on targeted transposition of three non-LTR (long terminal repeat) retrotransposons [295], which reinforce the suggestion that a retrotransposon gene was “domesticated” for a cellular role early in the evolution of eukaryotes [1]. On the other hand, *Allium* species lack any known telomeric sequence [296]. Instead, the chromosomal ends of *Allium* species consist of satDNA [297], which suggest a functional role for satDNA in telomeric function, at least in such exceptional cases (see below). 

Subtelomeric heterochromatin is composed of tandem repeats with a variety of lengths and repeat numbers [41,298]. However, it has been described some cases of high level of sequence conservation between species [42]. Subtelomeric repeats have been reported in numerous plant and animal species. For example, the human subtelomere structure and variation has been extensively analyzed [299,300]. In rye, several satDNA families have been described with different repeat lengths and great variability in organization in the subtelomeric heterochromatin [301,302,303,304]. CL14 and CL34 repeats show a complex satellite repeat composition of subtelomeres through *Solanum* species [305] as well as khipu and jumper satDNAs at subtelomeres of *Phaseolus* [306]. Subtelomeres are fast evolving regions [304,307]. Many subtelomeric satDNA sequences are species-specific, often chromosome-specific. The existence of several subtelomeric satDNA families or even subfamilies in one species is also common [301,308]. Rearrangements by recombination between non-homologous chromosome ends may lead to the formation of new satDNA families [91,305]. 

Frequently, short and often degenerate tracts of telomeric repeats are interspersed within the subtelomeric region and even occasionally within the single copy sequences adjacent to the subtelomeric repeats [27,29,47,300,309,310]. Interstitial telomeric repeats can also be found at centromeres as evolutionary relics derived from chromosomal rearrangements [27,311], although telomeric repeats invaded and were amplified within the functional centromeres of *Solanum* species [312].

Several functional roles has been proposed for subtelomeric DNA [298,300,313,314]: (a) genome stability; (b) faithful chromosome replication; (c) faithful chromosome pairing and segregation; (d) cell cycle regulation; (e) cellular aging and immortalization; (f) movements and localization of chromosomes within the nucleus; (g) transcriptional regulation of subtelomeric genes; (h) buffer terminal genes against the dynamic processes of loss and adition of telomeres. In addition, subtelomeric repeats may have an incidental role in cases of losing of conventional telomeric repeats. In such cases, chromosomal ends have been stabilized by subtelomeric satDNA [297,315]. In any case, as mentioned, these roles are sequence-independent. Further, it has been demonstrated that telomere-like sequences interspersed within subtelomeric DNA may play a role in subtelomeric recombination and transcription, in telomere maintenance via the alternative lengthening of telomeres (ALT) pathway, and in telomere healing [300,316,317].

Telomeric repeat-containing RNAs (TERRA) are long non-coding RNAs with sizes ranging between 100 nt and 9 kb, transcribed from telomeric C-rich strand of telomeres, initiating in subtelomeric regions, thus containing subtelomeric and telomeric sequences [233,318,319]. These RNAs were detected first in human and rodent as nuclear restricted RNAs that showed high levels in all adult tissues with low or no telomerase activity while showing low levels in cancer cells [318,319,320]. TERRA RNAs remain associated to telomeric chromatin as components of mammalian telomeres where negatively regulate telomere length and are involved in shortening of telomeres. TERRA transcripts have been detected associated with heterochromatin marks typical of telemores (H3K9me3) as well as with different telomere proteins including heterochromatin protein 1 (HP1) and the telomere repeat factors 1 and 2 (TRF1 and TRF2), among others, thus playing an important role in the maintenance of telomeric heterochromatin [233,321]. Over-elongation of telomeres leads to synthesize longer TERRAs which are involved in the recruitment of histone methyltransferases with the subesquent histone H3 methylation and the recovery of HP1, which in turn induce the transcriptional repression of TERRAs, preventing the heterochromatin hyperformation [318,319,320,321,322]. TERRA is thus involved in heterochromatin establishment but also has a role in the regulation of telomere length, in telomere capping and replication [233]. TERRA RNAs are evolutionarily conserved [319]. They have been described in different vertebrates [320] and yeast [323], as well as in *Arabidopsis* [324]. A portion of TERRA transcripts of *Arabidopsis* are processed into siRNAs, which promote methylation of cytosines in telomeric repeats and contribute to the maintenance of telomeric chromatin [324].

#### 3.5.3. Heterochromatin Assembly

Heterochromatin plays an essential role in preservation of epigenetic information, transcriptional repression of repetitive DNA, and proper chromosome segregation [72]. Even in the event of the difficulties for its analysis, today there is a great accumulation of data revealing the importance of heterochromatin in those roles. Specifically, the role of pericentromeric and subtelomeric satDNA transcripts is being brought to light.

Transcription from pericentromeric repetitive sequences has been reported in a number of plants and animals [71,72,239,325]. Small 20–25 bp long RNAs derived from centromeric DNA repeats of the fission yeast *S. pombe* are involved in heterochromatin assembly by the means of RNA interference (RNAi) processes [326]. For heterochromatin establishment, which in turn leads to a transcriptionally silent state, only low level of expression of repetitive DNA is necessary. This silencing mechanism of the chromatin is accomplished by RNA-induced transcriptional silencing complex (RITS) built by the association of Argonaute protein and siRNAs. Interestingly, these siRNAs are derived from the processing by Dicer of double-stranded RNA that arises from bidirectional transcription of repeated centromeric DNA [326]. RITS complex contributes to the formation of pericentromeric heterochromatin, whose establishment is necessary for proper chromosomal segregation in mitosis and meiosis [326]. Formation of heterochromatin also requires histone H3 methylation at lysine 9 (H3K9me2), essential for the recruitment of heterochromatin protein 1 (HP1). This promotes silencing gene expression, recombination, kinetochore assembly, and prevention of erroneous microtubule attachment to the kinetochores [72,326]. The RITS complex uses base pairing interactions between the loaded siRNA and nascent transcripts (along with H3K9me2 interactions) to localize to pericentric regions. Localized RITS complexes recruit histone methyltransferases, leading to H3K9 methylation, HP1 recruitment (Swi6 in *S. pombe*), heterochromatic silencing, and generation of more siRNAs through recruitment of Dicer and an RNA-dependent RNA polymerase (RdRP) [327,328]. In this way, heterochromatin is maintained through a self-reinforcing positive feedback loop; siRNA promotes H3K9 methylation which promotes siRNA production, and both siRNA and H3K9me2 interact with the RITS complex to facilitate its localization to pericentric heterochromatin [327,328].

Like in fission yeast, small RNAs contribute to heterochromatin regulation in fungi, ciliates, plants, and worms acting through RNA-dependent RNA polymerase (RdRP) , self-reinforcing feedback loops and histone methylation [327,328]. In plants, in addition to histone methylation, siRNAs also direct the methylation of the DNA from which they derived [329]. The regulation of heterochromatin assembly by siRNAs of 21–24 nt derived from satDNAs has been proven in several plant species such as *Arabidopsis*, rice, and sugar beet [265,329,330]. However, the involvement of RNAi in heterochromatin formation in other eukaryotic organisms is still debated [72,233]. Dicer and Argonaute protein families, components of the RNAi machinery, have been identified in the genome of the beetle *Tribolium castaneum*, but not the RdRP [72] which appearently has been lost in most insects and vertebrates [328]. Evidence for the regulation of heterochromatin assembly through an RNAi mechanism has been found in chicken [331], in *C. elegans* [332], in *Drosophila melanogaster* [333], and in mouse [334]. However, it is not still clear how heterochromatin regulation is mediated in these organisms. It appears that siRNAs are not involved in heterochromatin remodeling in mammals. However, it appears that a RNA is required but this RNA is still unindetified [335]. An RNAi-dependent mechanism that involves telomerase reverse transcriptase in heterochromatin establishment and maintenance has been recently proposed [233,336]. 

A relation between misregulation of centromeric and pericentromeric satDNA transcription and tumors has been established. De-condensation of pericentromeric heterochromatin and over-expression of pericentromeric satellite repeats is characteristic of some tumors and genetic disorders [72,233,325,337,338,339]. Three different studies revealed the overexpression of pericentromeric satDNAs in human tumors [325,337,338]. For example, the pericentromeric human satellite II (HSATII) is aberrantly overexpressed in a wide variety of epithelial cancers [338]. Induction of HSATII transcription is triggered by growth of cells under nonadherent conditions. The HSATII transcripts are reversely transcribed and this RNA-derived DNA is reintegrated with the subsequent expansion of HSATII loci [239]. HSATII copy number gain is a common feature in primary human colon tumors and is associated with a lower overall survival. Thus, cancer-associated derepression of specific repetitive sequences can promote their RNA driven genomic expansion, with potential implications on pericentromeric architecture [239]. Further, this study illustrates the point that satDNA profile might evolve rapidly in a species by retrotransposition of some satellites over others, at least under certain circumstances such as in the setting of cancer.

On the other hand, chromosomal instability induced by overexpression of α satellite transcripts appear involved in the development of breast and colorectal cancers [340,341,342]. Thus, although some alpha satellite transcription may be necessary for proper centromere and/or heterochromatin function, overexpression may have damaging effects [328].

#### 3.5.4. Gene Regulation

Promoter elements and transcription start sites, as well as binding motifs for transcription factors, have been mapped within some satellites which could influence nearby genes, probably by the mechanism of transcriptional interference [72,343].

Human satellite III DNA is transcribed in response to stress [72,233,344]. In human cells, the heat shock response is characterized by the transcriptional activation of both protein coding heat shock genes and noncoding repeated satellite III DNA sequences located at pericentromeric heterochromatin [345]. The transcriptional activation of sat III by the HSF1 protein (Heat Shock Factor 1, a transcription factor present in the nucleus and cytoplasm of cell before heat shock) could represent a powerful way to trigger a rapid remodeling of gene expression at a genome-wide scale in heat-shocked cells [345]. In addition to heat shock, various cellular stresses may also influence the expression of satDNA [233]. In humans, an example of gene expression mediated by heterochromatin transcripts is found in the testis-specific RNA transcripts from the MSY distal heterochromatic block of the Y chromosome which are involved in trans-splicing with CDC2L2 kinase mRNA generating a testis-specific isoform [71,346]. 

On the other hand, PRAT and PSUB satDNAs of beetles of the genus *Palorus* are differentially expressed depending on developmental stages and tissue types [258,268]. In such a way, transcription of many satDNAs have been found to depend on development during which satDNA transcripts may act as epigenetic signals required for centromere stability [347,348] and organization of pericentromeric heterochromatin, and might be necessary for differentiation and developmental progression [72,233].

Thus, several examples suggest that transcripts from satDNA might regulate gene expression in particular cases [72,233,327]. However, there are few experimental studies proving it. Nonetheless, there are two recent studies that demonstrate the specific role of siRNAs derived from processed noncoding RNAs transcribed from satDNA. The TCAST1 satDNA of the beetle *Tribolium castaneum* comprises the pericentromeric heterochromatin but is also dispersed within euchromatin [21,128,130]. Heat shock induces TCAST1 expression. Transcripts of this satDNA are long double-strand transcripts that are processed into 21–30 nt siRNAs which trigger an increase in repressive epigenetic modifications of histones at satDNA regions in heterochromatin [130,349]. Feliciello et al. [130] have demonstrated that short stretches of euchromatic TCAST1 satDNA loci modulate gene expression by enhanced suppression of activity of TCAST1-associated genes and slower recovery of their activity after long-term heat stress. The level of gene suppression is stronger as the number of copies of TCAST1 repeats is higher. The gene suppression consists in heterochromatin assembly mediated by siRNAs at euchromatic TCAST1 loci and surrounding regions. These authors found differences in the pattern of distribution of TCAST1 elements between populations which according to their proposal might contribute to gene expression diversity among populations of this species. Furthermore, they proposed that this diversity might have an adaptative impact on populations to different environmental conditions. Vlahović et al. [108] have proposed that HORs could act as gene regulatory elements and that variation in HOR composition among individuals or populations can generate gene expression diversity and contribute to the evolution of gene regulatory network. The second example focus in the control of dosage compensation in *Drosophila melanogaster* males, in which satDNA directs male-specific gene expression [350]. The resulting imbalance in gene dosage between males and females in *D. melanogaster* is compensated by increased expression from the single X chromosome of males. Processed siRNAs from transcripts of a family of X-linked satDNA repeats promote X recognition [351]. Joshi and Meller [352] have demonstrated that these small noncoding RNAs generated from X-linked satDNA repeats are *cis*-acting elements that guide dosage compensation machinery preferentially find X sequences. The siRNA pathway therefore promotes recognition guided by chromosome-specific repetitive sequences [352].

In addition, Hall et al. [353], have demonstrated also the crucial role of human HSATII satDNA in gene regulation. Small blocks of HSATII are found on the pericentromeres of 11 human chromosomes, but chromosome 1, and to a lesser extent chromosome 16, carries very large (~5–6 Mb) blocks of this ~26-bp tandem repeat. This study provides evidence for the biological significance of HSATII. They postulate that it may have a normal function during development, such as global epigenomic programming in early development, or gametogenesis. This proposal is based on the capacity of both HSATII DNA and RNA to amass and sequester accumulations of two epigenetic factors that regulate heterochromatin, the polycomb group (PcG) complex PRC1 (protein regulator of cytokinesis 1), and the MeCP2 (methyl-DNA binding protein 2). In cancer, PRC1 bodies form on the demethylated HSATII of the mega-satellite in chromosome 1, while MeCP2 bodies form on HSATII RNA, potentially leading to further changes in the epigenome. Thus, HSATII DNA and RNA can bind and impact distribution of chromatin regulatory proteins, which goes awry in cancer. In altered cells, two types of cancer-specific nuclear bodies are formed, which is caused by locus-specific deregulation of HSATII. The study indicates that HSATII demethylation not only may be caused by, but may contribute to epigenetic instability in cancer, possibly providing a survival advantage (e.g., a bigger ‘sponge’ for PcG proteins) [353].

## 4. Concluding Remarks and Perspectives

“An evolving topic” has tried to show the evolution of the concept of satellite DNA through time since its discovery in the early 1960s until today. The cause of this evolution has been the continuous advance of the technology used to address the issue. Although the definition of satDNA as highly repetitive DNA organized in tandem is still valid, nowaday we have a wider view of the term and we know that the concept includes a large variety of highly variable sequence types in terms of nucleotide composition, length and structure of repetitive units, organization, and localization. The effort of many researchers over these years has allowed us to better understand various aspects of the very nature of the diversity of satDNA sequences but also of the evolution of satDNA and of the multiple factors influencing it. The approach to the study of satDNA from a genomic perspective has also helped us to better understand the complete set of satDNA families composing the genome of eukaryotic species and their evolution, as well as to know the relationships between the different satDNA families and between chromosomes. On the other hand, satDNA, initially considered ‘junk’ DNA, the largest ‘garbage’ among the ‘rubbish’ that supposedly constituted the plentiful panoply of types of repeated DNA sequences in the eukaryotic genome [41], is now revealed as a part of the genome that has important implications in chromatin regulation as well as in gene regulation, and which plays important structural roles in vital functions, such as segregation or preservation of the genetic material.

After a few years in the early 21st century in which the technique relegated ostracism to the advance of knowledge about satellite DNA, today we can be optimistic and believe that both the genomic approach and the most advanced technology in the field of genes and genomics regulation will soon allow us to find out additional, even unknown, details of this fascinating part of the eukaryotic genome. The advance in the knowledge of functional roles of satDNA as well as its role in cancer onset and progression will be of special interest.
